# Pitch accent speakers exhibit speech-to-song transformation accompanied by reduced musicality ratings: a cross-linguistic study

**DOI:** 10.1007/s00426-026-02262-0

**Published:** 2026-02-28

**Authors:** Makiko Sadakata, Martha Nobbe Smyth, Marijn van ’t Veer, Akihiro Tanaka

**Affiliations:** 1 Institute for Logic, Language and Computation / Musicology Department, University of Amsterdam, Science Park 107, Amsterdam, 1098XG The Netherlands; 2 Research Master Brain and Cognitive Sciences, University of Amsterdam, Amsterdam, The Netherlands; 3 Leiden University Centre for Linguistics, Leiden University, Leiden, The Netherlands; 4 Department of Psychology, Tokyo Woman’s Christian University, Tokyo, Japan

## Abstract

The Speech-to-Song (STS) transformation is a phenomenon where repeated listening to speech sounds leads listeners to perceive them as increasingly musical. Previous research suggests that the STS transformation may be less pronounced for tonal language speakers when listening to speech in their native language. This study investigates whether a similar pattern is observed in speakers of Japanese, a pitch-accent language. The results reveal that native speakers of Japanese experience a degree of STS transformation comparable to that observed in Dutch and Hiberno-English speakers, yet show substantially lower overall songlikeness ratings for Japanese stimuli. We interpret these findings as consistent with a language-specific influence associated with Japanese. More broadly, the results contribute to our growing understanding that the function of pitch in one’s native language may play an important role in shaping how the effect manifests.

**Supplementary Information:**

The online version contains supplementary material available at 10.1007/s00426-026-02262-0.

## Introduction

Repetition has a peculiar effect on inducing musical qualities in our auditory experiences of acoustic stimuli (Margulis, 2013). For example, when the sound of dripping water is looped, it reveals musical qualities that are otherwise imperceptible in everyday contexts (Simchy-Gross & Margulis, 2018). Similarly, looping a speech fragment can make it sound like a song, a phenomenon known as the Speech-to-Song (STS) transformation (Deutsch et al., 2011). The STS transformation has been replicated in a variety of languages, including French, German, Dutch, and Italian, and more, with both robust effects and, in some cases, language-specific reductions (e.g., Jaisin et al., 2016; Falk & Rathcke, 2010; Groenveld et al., 2020; Rathcke et al., 2021). This transformation provides valuable insights into the mechanisms by which the brain categorizes incoming sounds as either musical or linguistic. Previous studies have investigated the acoustic characteristics of stimuli that contribute to the STS phenomenon (Falk et al., 20142014; Hiemstra and Sadakata 2024; Tierney et al. 2018a) and the role of various cognitive functions (Castro et al. 2018; Groenveld et al. 2020; Kubit et al. 2024; Rowland et al. 2019; Soehlke et al. 2022; Tierney et al. 2018b), as well as individual listening experiences (Jaisin et al., 2016; Kachlicka et al., 2024; Margulis et al., 2015; Tierney et al., 2021; Vanden Bosch der Nederlanden et al., 2015). This paper builds upon the latter line of research by examining the influence of the listener’s native language on their perception of the STS transformation.

Two distinct types of interactions between the native language and the STS transformation have been identified. The first type of interaction between native language and the STS transformation concerns language familiarity: stronger transformations are observed for unfamiliar languages, while familiar languages tend to produce weaker effects (Margulis et al., 2015).

The second type highlights how the type of one’s native language, particularly its use of pitch information, can significantly influence the transformation effect. Jaisin et al. (2016) compared Mandarin, Thai, German, and Italian speakers in terms of their STS experience in response to speech stimuli from those same four languages. They found that while all stimuli elicited the STS transformation, the effect was significantly attenuated for tonal language speakers (Mandarin and Thai) compared to non-tonal speakers (German and Italian). However, more recent findings suggest that the idea that tonal language speakers show a generally reduced STS effect may be too simplistic. Kachlicka et al. (2024) found that tonal and non-tonal speakers did not differ in their STS responses when listening to English stimuli, indicating that the interaction between language background and STS is more nuanced. Other work (Sadakata, 2024) suggests that while the STS transformation still occurs in tonal language speakers, its strength is attenuated when the stimulus is in their native language.

Although the mechanisms underlying this selective reduction remain to be fully understood, these findings support the idea that linguistic familiarity and pitch-processing mechanisms interact with acoustic repetition to shape the STS experience. Investigating speakers of pitch-accent languages in this context is particularly informative. Language such as Japanese lie between tonal and non-tonal languages in their use of pitch for linguistic processing, and therefore expected to provide critical insighs into the mechanisms underlying the STS transformation.

Understanding how pitch is used in language is essential for this study. Non-tonal languages, such as English and Dutch, use pitch for intonational and pragmatic purposes, such as signaling emphasis and questioning, but not for distinguishing individual word meanings. In tonal languages such as Mandarin Chinese and Thai, pitch contours are assigned to every syllable, and these patterns convey lexical meaning. As a result, changing the pitch pattern of a syllable can entirely change the meaning of a word. The current study focuses on Japanese, which is typically classified as a pitch-accent language. Unlike tonal languages, where most syllables carry a specific tone, pitch accent in Japanese operates over sequence of morae, the syllable-like timing units in Japanese. In Standard Japanese, each mora serves as a tone-bearing unit and is specified as high or low, but lexical contrasts arise from the relative pitch pattern across the word (or pitch phrase), rather than from independent tonal categories on individual syllables (Kubozono, 2018). For example, the word *ame* with a High–Low pitch contour means ‘rain,’ whereas *ame* with a Low–High contour means ‘candy’ in standard Tokyo Japanese, a minimal pair distinguished by pitch pattern.

Importantly, the connection between pitch accent and lexical meaning in Japanese allows for substantial variation. The same word may have different pitch patterns across dialects, and distinctions based on pitch in one region may not exist in another. Accent placement also varies substantially across speakers (Kawahara, 2015). An interesting recent example is the pronunciation of the Japanese era name introduced in 2019, ‘Reiwa,’ which has been realized with different pitch patterns, such as HLL and LHH. In addition, corpus-based research highlights the complexity and underspecification in mapping sound to meaning in Japanese. For example, Amano and Kondo (2000) note that, because a single word can have multiple, context-dependent meanings, their lexical database does not distinguish homophones by meaning or grammatical category if they share the same written and spoken form, as handling such complexity would make database construction unfeasible. Studies examining actual pitch height further challenge the view that pitch patterns in Japanese words directly convey lexical meaning. For instance, Sugiyama (2008) showed that final-accented and unaccented words are often acoustically indistinguishable when produced in isolation. However, when these same words are embedded in a sentence and followed by a particle, clear pitch cues, such as F0 rise, peak, and fall, re-emerge, allowing listeners to perceive the accent contrast. This suggests that the realization and perception of pitch accent in Japanese depend not only on the word itself but also on the prosodic context in which it appears. Pitch-related distinctions are therefore recovered through an interaction between word-level representations and surrounding intonational structure, rather than being fully specified at the level of isolated lexical items.

Such specific characteristics of pitch patterns in Japanese may foster a distinct sensitivity to pitch information. An ERP study by Tamaoka et al. (2014) found that when native Japanese speakers listened to sentences containing incorrectly pitch-accented words, their brain responses did not show the typical N400 effect—a neural marker commonly associated with difficulty integrating the meaning of a word into its sentence context, a semantic mismatch (Kutas & Hillyard, 1980). The absence of an N400 in Tamaoka et al.’s study suggests that Japanese listeners were able to process sentence meaning even when pitch accents were incorrect. This has been interpreted as evidence that Japanese speakers do not always rely on pitch accent to interpret word meaning during comprehension—likely due in part to the variability in accent placement and its relatively limited lexical role compared to tonal languages.

However, this finding should not be taken to imply that Japanese listeners are generally insensitive to linguistic pitch. On the contrary, previous research has shown that they do use pitch contour as an important cue for recognizing word meaning (Cutler & Otake, 1999). Furthermore, a range of studies suggests that sensitivity to pitch contour emerges early in development and is shaped by language-specific experience. For example, infants acquiring non-tonal languages, pitch is progressively down-weighted as a cue for lexical contrast, despite preserved sensitivity to pitch as an acoustic dimension (Mattock & Burnham, 2006), while Japanese-learning infants have been shown to continue to treat pitch contours as linguistically relevant at 14 to 18 months of age (Mugitani et al., 2019). In adulthood, language-specific weighting of pitch cues remains essential. Native Japanese listeners have been shown to rely on pitch-contour information, particularly F0 movement patterns, when interpreting lexical contrasts in Japanese, whereas listeners from non-tonal stress-accent languages such as English are less likely to treat these pitch contours as functionally contrastive, despite being perceptually sensitive to them (Shport, 2015). This asymmetry reflects differences in how pitch is mapped onto linguistic categories, rather than differences in basic pitch perception. Consistent with this interpretation, Japanese pitch-accent patterns are hard for English listeners to acquire, and even with training, the mapping between English stress accent and Japanese pitch accent remains non-trivial, highlighting that a direct one-to-one comparison between the two systems is not straightforward (Shport, 2016).

Taken together, these findings suggest that, although Japanese is not a tonal language, it promotes a distinct use of linguistic pitch information through its pitch-accent system. This makes Japanese a valuable case for examining how language-specific pitch experience shapes the STS transformation.

To investigate the impact of pitch systems in the native language on the STS transformation, we conducted an experiment involving native speakers of Japanese, Dutch, and English. Dutch participants were included because they were the most accessible group for the research team mainly based in the Netherlands. In addition to serving as a control group to confirm the general STS transformation for both types of stimuli, they were also involved in several pilot studies to assess the potential influence of test locations and methods.

Before we proceed, it is helpful to clarify how the STS transformation is typically operationalized in the literature and how we approach it in this study. Although the term “speech-to-song” suggests a categorical shift, listeners do not always report hearing a full song. Instead, the transformation is often described as a subtler increase in perceived musicality—such as a greater sense of melody or songlikeness—without a complete reclassification into the musical domain. This graded view is reflected in much of the empirical literature on STS. For example, Deutsch and colleagues (2011), who introduced the phenomenon, demonstrated that repeated exposure to certain speech fragments led to higher songlikeness ratings using a 5-point Likert scale, ranging from 1 (“exactly like speech”) to 5 (“exactly like singing”). Many subsequent studies have adopted similar rating-based methods, treating the STS transformation as a continuous shift in perceived musicality rather than a binary change. In our study, we follow this behavioral tradition and operationalize the transformation as a significant increase in perceived songlikeness across repeated presentations of a speech fragment, measured using a 7-point Likert scale.

While previous studies have examined the potential influence of musical training on the STS illusion, there are no consistent findings indicating an association between musical expertise and the strength of the STS effect (Falk et al., 2014; Vanden Bosch der Nederlanden et al., 2015). Smit and Rathcke (2024) reported that musical training does not independently influence the experience of the illusion, although it may interact with linguistic parameters, such as syntax complexity. Additionally, Tierney et al. (2021) found that perceptual aptitude for musical sounds, rather than musical training per se, is a more reliable predictor of the experienced STS transformation. These together suggest that the broad measure of musical training may not capture the core factor related to the STS transformation. While investigating the effect of musical training was not the primary goal of our study, we administered the Musical Training subscale of Goldsmiths Musical Sophistication Index (Golds-MSI) because this information is informative for facilitating comparison with other studies. We used the validated Japanese version (Sadakata et al., 2023), the original English version (Müllensiefen et al., 2014), and an unpublished Dutch translation adapted for use in prior local research projects.

Participants were asked to rate the songlike qualities perceived in speech segments presented once (initial presentation) and after repetition (final presentation), in both Japanese and English. We had three main predictions. First, we expected to replicate the classic STS transformation, with songlikeness ratings increasing from initial to final presentations. Second, we predicted that participants would give lower songlikeness ratings for their native language compared to a non-native language. Third, we hypothesized that Japanese participants’ responses would be different from non-tonal speakers. Specifically, based on prior findings with Mandarin Chinese speakers (Jaisin et al., 2016)—who show almost no STS transformation for their native language—we expected Japanese participants to show a strong transformation for English stimuli but a smaller effect for Japanese stimuli, reflecting their pitch-accent background. We evaluated these effects both in terms of the absolute songlikeness rating values (how songlike the speech was perceived) and the transform score (how strongly the fragment was perceived to transform from speech to song).

## Methods

### Recruitment

Participants were recruited via convenience sampling within the academic and local social circles of the research team, including students, colleagues, and acquaintances in Japan, the Netherlands, and Ireland. All testing sessions were conducted using the same testing platform; for a subset of Dutch participants (*n* = 12), a researcher was present during their participation to validate the testing procedure (see “Dutch participant data used for methodological validation” for details). All participants volunteered to take part and did not receive monetary or material compensation.

### Participants

We recruited native speakers of three language groups: 17 Japanese, 29 Dutch, and 20 Hiberno-English speakers. All Japanese data were collected from participants residing in Japan; one native Japanese speaker was excluded for not meeting this criterion. Data from nine Dutch participants were excluded because they had participated in the same experiment using a different rating scale (5-point Likert scale). Please refer to the detailed inclusion and exclusion criteria for Dutch participants provided in the “Dutch participant data used for methodological valication” section below the Methods.

The final analysis included data from 55 participants divided into three groups: 16 native monolingual speakers of Japanese (JP) in Japan, 19 native monolingual speakers of Hiberno-English (EN) in Ireland, and 20 multilingual speakers of Dutch (DU) in the Netherlands. Table 1 describes the summary of language bacgkround and musical training characteristics for the three groups.

**Table 1:** Summary of Language Bacgkround and musical training characteristics for the Japanese, Dutch and english particiapnt groups. Values are reported as mean (SD) unless otherwise specified. Self-rated english proficiency and frequency of english use are based on 5-point likert scales, with scale anchors indicated in the table. Gold-MSI scores refer to the musical training subscale of the goldsmiths musical sophistication index

Measure	Participant groups		
	Japanese	Dutch	English
Sample size	16	20	19
Participants with fomal English training (n)	13	19	n.a. (native)
Time since last English instruction (years)	0–20	0.5–12	n.a. (native)
Self-rated English proficiency (1: beginner, 2: intermediate, 3: advanced, 4: fluent, 5: native-like)	1.23 (0.44)	M = 3.9 (0.66)	n.a. (native)
English use – speaking (1: everyday, 2: once a week, 3: once a month, 4: less than once a month, 5: never)	4.15 (0.99)	M = 1.26 (0.65)	n.a. (native)
English use – listening (1: everyday, 2: once a week, 3: once a month, 4: less than once a month, 5: never)	3.23 (1.36)	M = 1.05 (0.22)	n.a. (native)
Formal Japanese training	n.a. (native)	None	None
Other conversational languages	None	German (6), French (4), Spanish (2), Swedish (1), Portuguese (1)	Irish (2), German (2)
Gold-MSI (musical training)	21.87 (12.89)	31.4 (8.44)	25.6 (10.09)

#### Japanese group (JP)

The JP group consisted of 16 native Japanese speakers (3 male, 13 female), with an average age of 26.1 years (SD = 3.61). English was a familiar but non-dominant language.

#### English group (EN)

The EN group consisted of 19 native speakers of Hiberno-English recruited in Ireland (8 male, 11 female), with a mean age of 23.6 years (SD = 2.47). None of the participants had received formal training in Japanese.

#### Dutch group (DU)

The DU group consisted of 20 native Dutch speakers (7 male, 13 female), with a mean age of 25.0 years (SD = 4.58). Their self-rated English proficiency was high and frequency of use was near-daily. None were fluent in Japanese (no one had followed any forms of lessons).

##### Stimuli

Thirty stimuli were selected for the experiment, designed to induce a heightened perception of songlikeness in both English and Japanese listeners. The English stimuli were adapted from Tierney et al. (2013) and consisted of speech fragments from audiobooks featuring three male speakers whose voices with British English accent were intended to resemble natural speech (stimuli AT0001 to AT0015). These stimuli had been previously validated to reliably induce the transformation.

The Japanese stimuli were recorded by a female speaker with a Tokyo standard accent. Initially, 12 speech phrases were recorded, each longer than three seconds. To match the shorter duration of the English stimuli, these phrases were segmented into 24 shorter clips. A pilot evaluation involving seven native English listneres was conducted to assess the degree of perceived STS transformation for each segment using the same task as in the current experiment (see Task section). The 15 segments that elicited the strongest tranfsomration from speech to songlike perception were selected for use.

To assess the comparability of the two stimulus sets, we examined key acoustic properties at the stimulus-file level. Each stimulus was segmented into syllable- or mora-like intervals, allowing for detailed analyses of total duration, average syllable duration, median F0, and pitch variability (mean F0 change per second in semitones). Because some variables violated normality assumptions, Wilcoxon rank-sum tests were used when appropriate.

The total duration per file did not significantly differ between the two sets (English: *M* = 1.23 s, *SD* = 0.35; Japanese: *M* = 1.39 s, *SD* = 0.25), *t*(27.83) = − 1.67, *p* = .107. Average syllable duration was also comparable (*W* = 154, *p* = .089). As expected based on speaker gender, the Japanese stimuli had a significantly higher median F0 (*M* = 259.9 Hz, *SD* = 80.6) than the English stimuli (*M* = 144.2 Hz, *SD* = 50.2), *W* = 13, *p* < .00001. However, pitch variability did not differ significantly (*W* = 124, *p* = .653), suggesting both sets involved similar levels of pitch modulation within each stimulus.

While the English and Japanese stimuli differed in speaker gender, number of voices, and median F0, these differences are unlikely to severely confound the main comparisons, as all participants were exposed to both stimulus sets. Our primary aim was to assess whether Japanese listeners exhibit a distinct perceptual response to Japanese stimuli, a pattern not expected in Dutch or English participants. This cross-language, within-subject design minimizes the risk that general acoustic differences systematically bias the observed effects and enables the identification of listener-specific responses.

### Procedure

The experiment was conducted using an online experiment platform Experiment Designer (Vet, n.d.). All participants completed the same experiment, with instructions provided in their native language (Japanese, Dutch, or English). Participants were instructed to wear headphones and complete the experiment in a quiet environment to ensure optimal audio quality and minimize external distractions.

Participants were informed that the study investigated how the language of the listener and the stimulus might influence the perception of the STS transformation, a phenomenon in which repeated exposure to a spoken phrase may cause it to sound songlike. They rated each stimulus on a 7-point scale from “most speech-like” to “most song-like.” No further explanation of these terms was provided; interpretation was left to the listener.

The experiment began with a single practice trial featuring the speech phrase “Sometimes behave so strangely”, taken from Deutsch et al. (2011). Following the practice trial, participants listened to 30 stimuli. Each stimulus was rated twice. The first rating was recorded after the stimulus was played once, and the second rating was provided after the stimulus had been repeated eight times. During the repetition phase, each stimulus was presented consecutively with a 400 ms pause between repetitions.

The 30 stimuli were organized into four blocks, each containing 7 or 8 stimuli. Within each block, stimuli were grouped by language (Japanese or English) for consistency. To counterbalance and minimize potential order effects, an ABBA presentation design was used, with stimuli presented in one of two sequences, randomly assigned: JP-EN-EN-JP or EN-JP-JP-EN.

### Dutch participant data used for methodological valication

We originally tested a group of 27 native Dutch participants under varying conditions. This group served both to validate decisions about testing location (online vs. onsite) and response format (5-point vs. 7-point Likert scale), and to collect data for potential inclusion in the final analysis. Twelve participants were tested onsite using a 7-point scale. The remaining 15 participated online: 9 used a 5-point scale, and 6 used a 7-point scale. The stimuli and overall methodology were held constant across conditions.

To evaluate the influence of testing location, we fitted linear mixed-effects models with initial and final rating as the dependent variables, including testing location (in-person vs. remote) as a fixed effect and subject as a random intercept. The models showed no significant effect of testing location on either initial (b = 0.77, *p* = .080) or final ratings (b = 0.30, *p* = .47), suggeting that remote testing did not meaningfully alter the results. In contrast, when comparing rating scales, final ratings collected using a 7-point scale were significantly higher than those using a 5-point scale (b = 0.23, *p* = .022), suggesting that the broader scale provided greater sensitivity to participants’ perceptual shifts.

Based on these findings, all other data collection was conducted online using the 7-point scale. To ensure procedural consistency across groups, only the 20 Dutch participants tested with the 7-point scale were retained for final analysis. We think that additional response options may allow participants to express finer distinctions in their judgments, potentially revealing behavioural patterns with greater sensivitiy.

### Statistical analysis

In the present study, Likert-scale ratings were analyzed using linear mixed models (LMMs), treating responses as quasi-continuous. This approach follows common practice in experimental psychology and allows us to model crossed random effects of both participants and stimuli. While this analytic choice is primarily methodological, recent theoretical work offers a useful perspective on how responses on Likert scales may be interpreted. Douven (2018), for example, argues that mid-scale response categories, often attributed to indecision or response bias, can instead reflect rational responding under uncertainty. Selecting extreme response options typically requires strong internal evidence, whereas such evidence may be weakened by factors such as stimulus ambiguity, competing interpretations, or limited confidence in one’s own judgment. In these situations, choosing a mid-scale response category constitutes a reasonable and informative response, rather than merely reflecting indecision or response bias, as it can express a moderate level of internal evidence. From this perspective, Likert-scale ratings can be interpreted as discrete expressions of underlying continuous internal estimates.

Statistical analyses were conducted in R (version 4.3.2; R Core Team, 2023) running on macOS Sonoma 14.3. Linear mixed-effects models were fitted using the lme4 package (Bates et al., 2015), and tests for fixed effects were performed using lmerTest (Kuznetsova et al., 2017). Estimated marginal means and post-hoc comparisons were computed using the emmeans package (Lenth, 2024). Model diagnostics were performed using DHARMa (Hartig, 2024). Data visualization was carried out using ggplot2 (Wickham, 2016) and gghalves (Tiedemann, 2022).

## Results

**Fig. 1:**
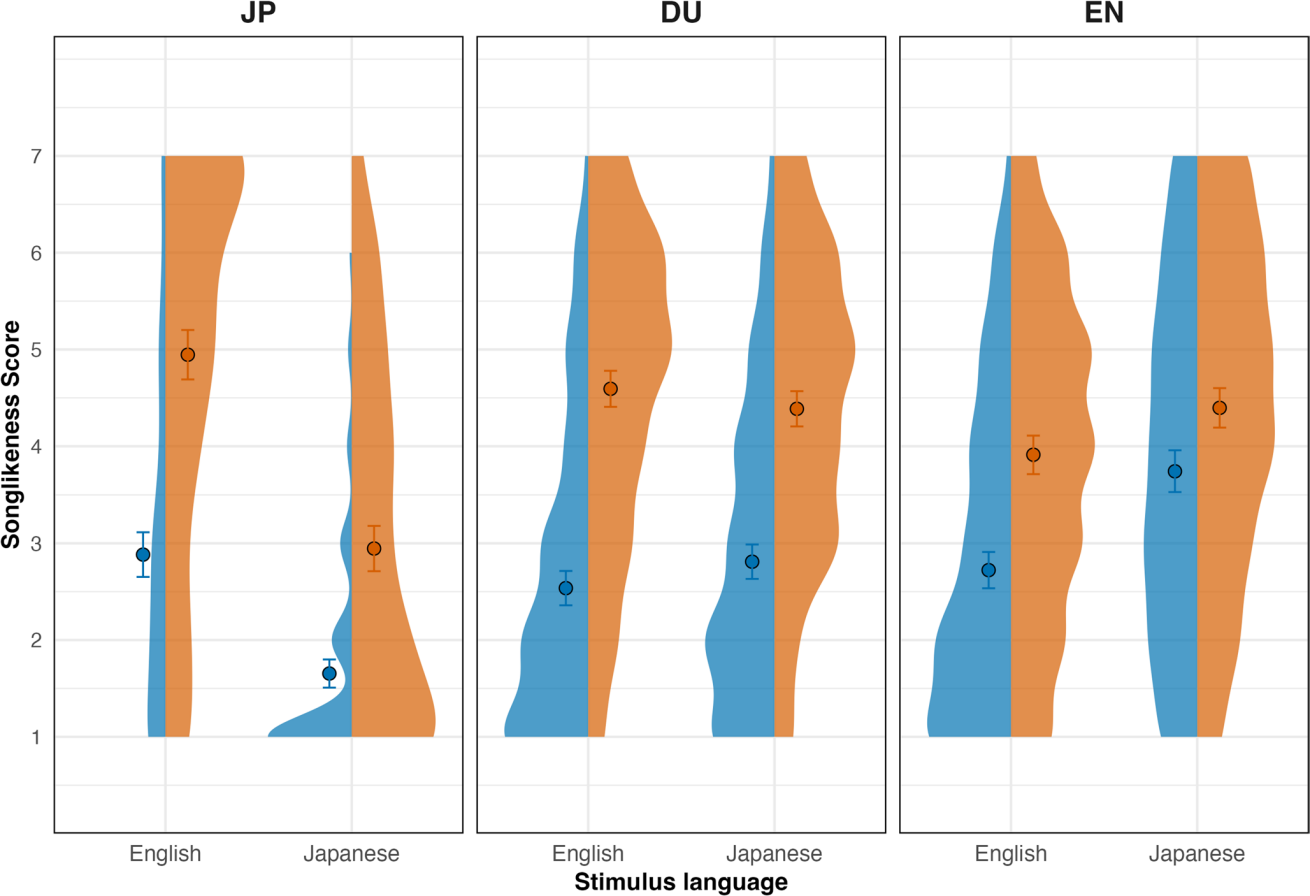
Plots of songlikeness ratings by group and stimulus language. For each participant group (JP, DU, EN), the distribution of songlikeness scores is shown for English and Japanese stimuli. Within each language, the left half of the violin reflects responses after the first presentation (Initial) and the right half reflects responses after repetition (Final). Dots indicate mean ratings for each condition, with error bars representing 95% confidence intervals. Ratings range from 1 (“most speech-like”) to 7 (“most song-like”)

Figures 1 illustrate the observed songlikeness ratings by group and stimulus language. A LMM was fitted to examine the effects of ResponseType (Initial vs. Final), group (JP, EN, DU), and Language (stimulus language: English vs. Japanese) on songlikeness rating. A random intercept for subject was included to account for repeated measures. The model reference levels were set to JP (Group), English (Language), and Initial (ResponseType). A full model, including three-way interactions between all predictors, was compared to a simplified model with only two-way interactions and main effects. The comparison showed no significant improvement in model fit for the full model, χ²(2) = 1.34, *p* = .51 (Supplementary Information, Table S1). The simplified model was retained for interpretability and parsimony. Model syntax, estimates, standard errors, degrees of freedom, *t*-values, and *p*-values are presented in Table 2. To test specific hypotheses, pairwise post-hoc comparisons were conducted using estimated marginal means with Tukey correction for multiple testing.

**Table 2:** Statistical details of the simplified model**.** Model syntax: Response ~ ResponseType * Group + ResponseType * Language + Group * Language + (1 | Subject). The table presents estimates, standard errors, degrees of freedom, t-values, and p-values for all fixed effects and interactions

Effect	Estimate [95% CI]	SE	df	t	*p*
(Intercept)	2.93 [2.51, 3.34]	0.21	67.50	13.87	< 0.0001***
ResponseTypeFinal	1.97 [1.75, 2.19]	0.11	3238.00	17.74	< 0.0001***
Group JP vs. DU	-0.41 [-0.97, 0.13]	0.28	65.42	-1.49	0.141
Group JP vs. EN	-0.22 [-0.78, 0.34]	0.28	65.42	-0.77	0.444
LanguageJapanese	-1.33 [-1.54, -1.10]	0.11	3238.00	-11.91	< 0.0001***
ResponseTypeFinal X GroupDU	0.1396 [-0.12, 0.40]	0.13	3238.00	1.07	0.287
ResponseTypeFinal X GroupEN	-0.75 [-1.02, -0.50]	0.13	3238.00	-5.70	< 0.0001***
ResponseTypeFinal X LanguageJapanese	-0.58 [-0.79, -0.38]	0.10	3238.00	-5.54	< 0.0001***
GroupDU X LanguageJapanese	1.65 [1.39, 1.90]	0.13	3238.00	12.57	< 0.0001***
GroupEN X LanguageJapanese	2.37 [2.11, 2.63]	0.13	3238.00	17.85	< 0.0001***

We evaluated model assumptions using simulation-based residual diagnostics implemented in the DHARMa package. These diagnostics indicated no evidence of over- or under-dispersion. Minor deviations from residual uniformity and a small number of extreme observations were observed, as expected for discretized Likert-scale data with the current sample size. Sensitivity analyses excluding extreme residuals yielded the same qualitative pattern of results. Diagnostic plots and a report of the sensitivity analyses are provided in the Supplementary Information (Figure S1).

The simplified model revealed a significant intercept (*b* = 2.93, *t*(67.5) = 13.87, *p* < .0001), indicating that Japanese participants gave an average songlikeness rating of 2.93 when listening to English stimuli for the first time (i.e., at Initial rating; the reference condition for both Group and Stimulus Language). There was a significant main effect of response type (*b* = 1.97, *t*(3238) = 17.74, *p* < .0001), reflecting that, for Japanese participants listening to English stimuli, Final ratings were on average 1.97 points higher than Initial ratings, demonstrating an increase in perceived songlikeness after repeated exposure. The main effect of stimulus language was also significant (*b* = -1.32, *t*(3238) = -11.91, *p* < .0001), such that, for Japanese participants, songlikeness ratings for Japanese stimuli were on average 1.32 points lower than for English stimuli (collapsed across response type).

There was no significant main effect of Group. JP participants did not differ from DU participants (*b* = − 0.41, *t*(65.42) = − 1.49, *p* = .141), nor from EN participants (*b* = − 0.22, *t*(65.42) = − 0.77, *p* = .444). However, the interactions with response type and stimulus language suggests distinct group-specific patterns. To further clarify the nature of the observed interactions, we examined each significant and non-significant term in the model.

The interaction between response type and group revealed that DU participants did not differ significantly from JP participants in how their ratings changed from initial to final presentations (*b* = 0.14, *t*(3238) = 1.07, *p* = .287). In contrast, EN participants showed a significantly smaller increase in ratings compared to JP participants (*b* = − 0.75, *t*(3238) = − 5.70, *p* < .0001), suggesting a weaker STS transformation in the EN group. A significant interaction between response type and stimulus language indicated that the increase in songlikeness ratings from initial to final (i.e., the STS transformation) was generally smaller for Japanese stimuli than for English stimuli, across participant groups (*b* = − 0.52, t(3238) = − 5.54, *p* < .0001).

To sum up so far, the model indicates an increase in songlikeness ratings from initial to final presentations, consistent with an STS effect. This increase was significantly larger for JP participants than for EN participants, while DU participants did not differ reliably from JP participants in the magnitude of the increase. Overall, the increase in songlikeness ratings was greater for English stimuli than for Japanese stimuli.

The Group × Stimulus Language interaction provided key insight into how listeners from each group responded to speech in their fluent versus non-fluent languages. Both DU and EN participants rated Japanese stimuli significantly more songlike than JP participants (DU: *b* = + 1.65, t(3238) = 12.57, *p* < .0001; EN: *b* = + 2.37, t(3238) = 17.85, *p* < .0001), indicating that reduced songlikeness ratings for Japanese stimuli were specific to JP participants.

To clarify this interaction, we examined group differences within each stimulus language using post-hoc pairwise comparisons of estimated marginal means (Tukey-adjusted). For English stimuli, songlikeness ratings did not differ significantly between JP, DU, and EN participants (all *p* > .40), indicating comparable evaluations of English speech across groups. In contrast, for Japanese stimuli, JP participants rated the stimuli significantly lower than both DU and EN participants (JP vs. DU: *b* = − 1.30, *p* < .0001; JP vs. EN: *b* = − 1.77, *p* < .0001), while DU and EN participants did not differ reliably from each other (*p* = .17).

**Fig. 2:**
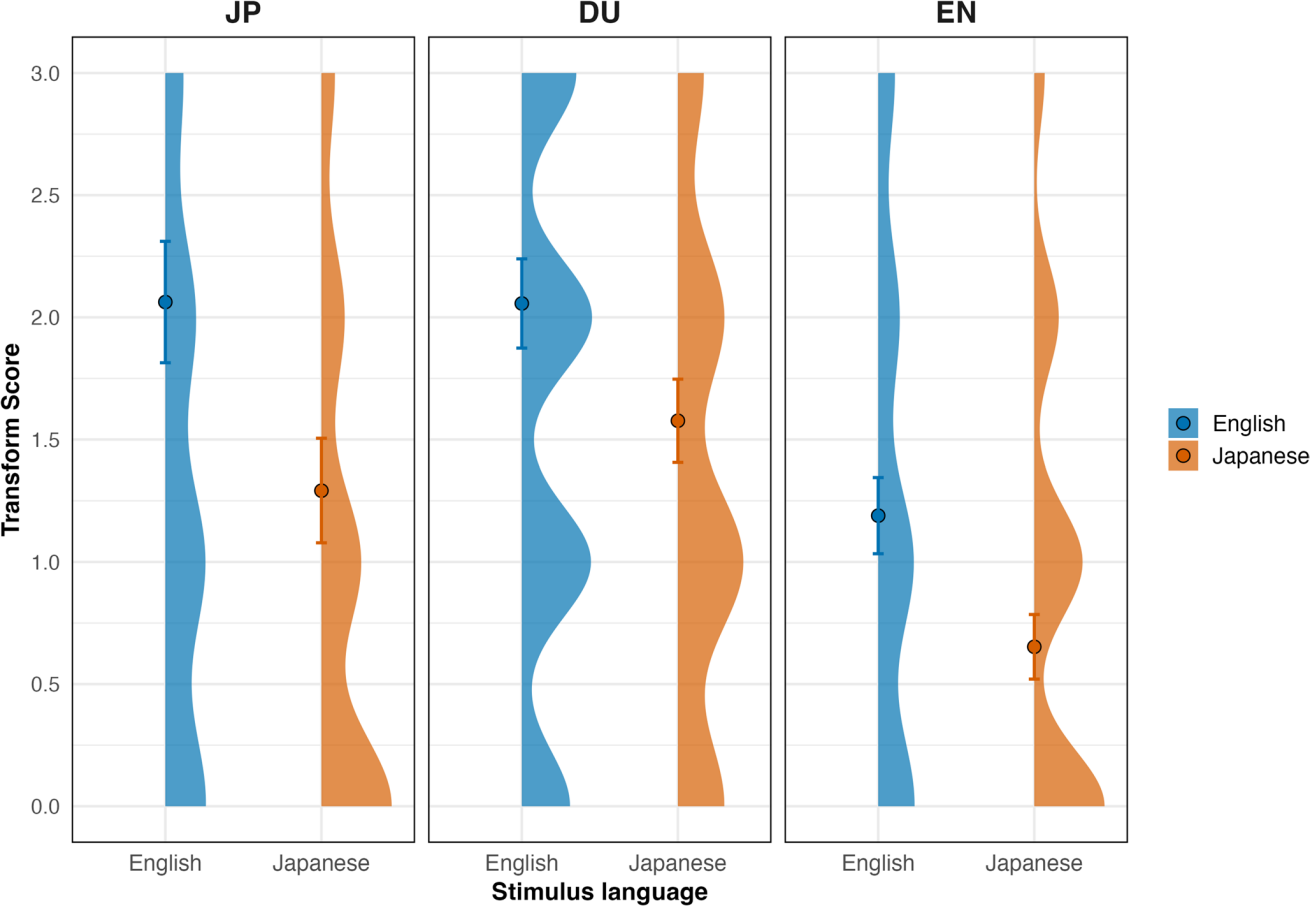
Plots of Transform Scores by group and stimulus language. For each participant group (JP, DU, EN), the distribution of Transform Scores is shown for English and Japanese stimuli. Each plot displays the right-side density of observed Transform Scores for the corresponding language and group. Dots indicate mean Transform Scores, with error bars representing 95% confidence intervals. The y-axis spans the range of observed scores

Although the Initial–Final difference was directly modeled in the above mixed-effects analysis, we additionally report Transform scores descriptively to summarize the magnitude of perceptual change and to facilitate comparison with prior STS studies that operationalize transformation as a difference score. Inferential conclusions are based on the mixed-effects model. Figure 2 provides the average Transform scores for the visual comparisons across groups and conditions.

Overall, Transform scores were positive across all groups and languages. Japanese and Dutch participants exhibited similar Transform scores across both English and Japanese stimuli, suggesting a comparable degree of perceptual change with repetition. This similarity in Transform magnitude occurred despite differences in absolute songlikeness ratings between the JP and NL groups, indicating that comparable levels of change can accompany different overall rating levels.

English participants showed smaller Transform scores overall reflecting a reduced increase in songlikeness rating with repetition relative to the other groups. Differences in Transform scores across languages were the smallest amont the three groups, with lower transformation magnitudes observed for Japanese stimuli than for English stimuli, consistent with patterns observed in the mixed-effects analysis.

### Effect of musical training

To assess whether musical training influenced songlikeness ratings or the STS transformation, we examined correlations between Golds-MSI scores and key outcome measures (Initial, Final, and Transform scores) separately by group and stimulus language. Because Gold-MSI scores showed different distributions across language versions, and because cross-language measurement invariance has not been formally established, direct comparison of musical training scores across groups would be difficult to interpret, as observed differences could reflect translation-specific or response-style effects rather than true differences in musical sophistication (Sadakata et al., 2023). Results are summarized in Table S2 (Supplimentary Information). Given the number of correlations examined, we applied the false discovery rate (FDR) correction, which balances control of Type I errors with sensitivity to true effects and is less conservative than the Bonferroni method.

Some positive correlations emerged between musical sophistication and songlikeness ratings, particularly for initial ratings in the Dutch group and for transformation scores among English participants listening to Japanese stimuli. For Dutch and Japanese participants, absolute songlikeness ratings tended to correlate positively with musical sophistication, whereas no clear or consistent pattern was observed for English participants.

## Discussion

### Language-specific modulation of STS

The current study investigated how native speakers of pitch-accented language (Japanese) respond to the STS transformation. The central question was whether their responses would represent a different pattern in comparison to speakers of a stress-accented language (Dutch and English).

In general, the results are consistent with our predictions. First, we replicated the STS transformation across all groups and conditions, with participants showing a significant increase in songlikeness ratings after repeated exposure to speech stimuli. Second, we found that songlikeness ratings tended to be lower for participants’ own native language, consistent with the idea that the STS transformation is modulated by linguistic familiarity (Margulis et al., 2015). Japanese participants rated English stimuli as more songlike than Japanese stimuli. In contrast, both Dutch and English participants rated Japanese stimuli significantly more music-like than Japanese participants did (Group × Language interaction), indicating that the suppression of songlikeness for Japanese speech was specific to Japanese listeners and was not observed in the other groups.

Third, and most critically for our main interest, Japanese speakers exhibited a STS transformation in their native language—comparable to non-tonal language speakers—yet their songlikeness ratings for Japanese stimuli were lower. Importantly, Japanese participants showed similar absolute levels of songlikeness ratings for English stimuli to those of Dutch and English speakers after repeated presentations, indicating that their lower ratings for Japanese stimuli are language-specific rather than reflecting a general reluctance to rate speech as musical.

Whether the reduction in songlikeness ratings for Japanese stimuli by Japanese listeners is larger or smaller than that reported in previous studies cannot be statistically tested here, as those studies were conducted independently. For context, previous work with English speakers found that unfamiliar, hard-to-pronounce languages were rated approximately one point higher than the native language after repetition on a five-point scale (Margulis et al., 2015). In the present study, the difference between Japanese and English stimuli for Japanese participants exceeded two points on a seven-point scale. In contrast to the extreme native-language suppression reported for tonal language speakers (Jaisin et al., 2016), Japanese listeners did not show such a complete attenuation of the STS effect.

Overall, these results support the idea that linguistic experience plays an important role in shaping the STS transformation (Castro et al., 2018; Margulis et al., 2015; Soehlke et al., 2022), and they highlight the need for further research examining how language-specific uses of pitch may influence the strength and characteristics of the STS transformation.

### Methodological considerations

One intriguing and somewhat puzzling observation in our data is the response pattern of our English speakers. Their initial songlikeness ratings were relatively high for both English and Japanese stimuli, resulting in comparatively low transformation scores. These results could reflect methodological artifacts or genuine effects associated with group characteristics. However, we are reasonably confident in the reliability of our methodological setup, as the same procedures were applied across all three groups. Thus, a more plausible explanation is that the results reflect characteristics specific to this group.

The English-speaking participants in this study were recruited from Ireland for practical reasons. At the time of recruitment, regional dialect was not expected to substantially influence the results, as previous work using the same English stimuli produced typical STS effects in Dutch participants, whose native language is typologically more distant from English than any regional variety of English (Groenveld et al., 2020). Hiberno-English, spoken in Ireland, reflects a blend of Irish and English influences and shows distinctive variation at multiple linguistic levels. Vocabulary and grammatical features set Irish English apart from other varieties (Dolan, 2006), and phonetic and prosodic characteristics further distinguish its sound pattern, including systematic variation in pitch height and contour patterns that are used to characterize regional and social variation, as well as stress timing (Hickey, 2008). Variation in syntax and the influence of social factors on language use are also well documented, underlining the complexity of Hiberno-English as both a regional variety and and a product of long-term contact between English and Irish (Kallen, 2012). Despite this variation, Hiberno-English remains a non-tonal, stress-accent language, and its prosodic structure does not involve lexical pitch contrasts. For this reason, dialectal variation within English was not expected to substantially influence the STS transformation observed for English stimuli in the present study.

Most English-speaking participants were recruited from the Dublin area, where pitch variation is generally moderate. While other segmental differences, such as vowel and consonant pronunciation, may still affect speech perception, regular exposure to these dialectal features could plausibly have contributed to the relatively high songlikeness ratings for English stimuli in this group. More generally, the potential influence of regional mismatch between stimulus speakers and listeners should be considered when interpreting these findings and in the design of future studies.

This study specifically focused on pitch-based typologies, motivated by both theoretical and empirical considerations. Prior research suggests that linguistic experience with pitch plays an important role in modulating STS perception (Jaisin et al., 2016; Kachlicka et al., 2024; Sadakata, 2024). Acoustic analyses of the STS illusion have identified repeated pitch contours as a key driver of the perceptual shift (Groenveld et al. 2020; Tierney et al. 2018a). However, temporal features have also been shown to contribute (Falk et al., 2014). Future studies could usefully compare languages that vary in temporal typology to further clarify the role of rhythm and timing in the STS transformation.

Several other aspects warrant consideration. The sample size in each group was modest, which may have limited the power to detect more subtle effects. The stimuli were not completely matched across languages; English materials were spoken by a male speaker and Japanese by a female speaker, introducing differences in pitch range and timbre. Future studies could aim to control for or systematically vary speaker characteristics, though creating a fully balanced set of STS-inducing stimuli that matches all relevant aspects across languages and speakers remains a significant methodological challenge. The full set of annotated stimuli used in this study is available for future research at https://osf.io/c37td. The current study did not include formal attention checks or post-task compliance questions; future studies could consider incorporating such measures to ensure data quality, particularly in online settings. Finally, the reliance on English listeners in the pilot evaluation may not fully capture perceptual responses representative of Japanese listeners, though our primary aim was to ensure that the selected Japanese speech segments could elicit the STS transformation under conditions and populations (non-Japanese listeners) where the effect is well documented.

### Cross-linguistic insights on the STS phenmoenon

The function of pitch in one’s native language may influence not only the perception of linguistic pitch, but also how repeated speech is abstracted and categorized musically. While further confirmation is needed, the present findings are in line with this view. This raises broader questions about how listeners process and organize auditory input based on their language background.

Classic studies of speech categorical perception (e.g., Harnad, 1987; Kuhl, 1991) emphasize how listeners acquire language-specific categories, compressing continuous acoustic variation into discrete linguistic distinctions. In parallel, more recent theories of auditory perception (McDermott et al., 2013) propose that the auditory system summarizes and compresses complex sounds into statistical representations based on prior experience. Together, these theories may shed light on how language experience shapes the transformation of speech into song and the boundaries between language and music.

For example, the tendency for non-native speech to be more readily transformed into song may arise because speech that lacks semantic meaning for the listener can be more easily compressed into summary statistics, such as pitch or loudness, making it more susceptible to musical interpretation. In contrast, when speech is familiar and meaningful, these abstraction processes may interact with language-specific coding mechanisms, such as semantic extraction and syntactic processing, which operate on well-established native phonetic categories, lexical tone representations, and prosodic patterns, thereby reducing the likelihood of musical transformation. In this way, linguistic experience may dynamically shape how listeners organize and interpret sound, influencing the switch between linguistic and musical interpretation of auditory inputs.

As such, cross-linguistic studies of the STS transformation combined with existing theories in linguistic and auditory perception may offer useful insights into how language experience shapes auditory perception at the boundary between speech and music.

### Future directions

Future research could address several further questions. First, direct comparison with speakers of other pitch-accent languages would help clarify whether the observed effects are specific to Japanese or reflect broader properties of pitch-accent systems. Second, experimental manipulations that alter the pitch salience or pitch contours of Japanese stimuli could test the boundaries of the observed suppression effect and identify the acoustic features most critical for triggering (or inhibiting) the STS transformation. Third, more detailed profiling of participants’ musical training, language proficiency, and exposure to other language varieties would clarify the individual differences underlying musicalization patterns. Finally, our findings point to the importance of not only broad language categories—such as tonal, pitch-accent, and non-tonal languages—but also finer distinctions within a language, such as regional accent or dialect. Future studies that systematically manipulate the match between listener and stimulus dialects could clarify the extent to which these within-language variations influence the perception of speech as song, particularly in English. These findings offer valuable insights into how pitch systems in one’s native language interact with the categorization of pitch information in repeated speech.

## Supplementary Information

Below is the link to the electronic supplementary material.

Supplementary Material 1 (DOCX 332 KB)

## Data Availability

The complete dataset, including formatted data, participant demographic information, stimulus files, sound annotations, and all analysis scripts, has been archived to the Open Science Framework (OSF; Sadakata, 2025) and can be accessed at [https://osf.io/c37td](https:/osf.io/c37td).

## References

[CR1] Amano, N., & Kondo, T. (2000). On the NTT database series lexical properties of Japanese [NTTデータベースシリーズ「日本語の語彙特性」について]. *Onsei Kenkyu (Journal of the Phonetic Society of Japan)*, *4*(2), 44–50. 10.24467/onseikenkyu.4.2_44

[CR2] Bates, D., Mächler, M., Bolker, B., & Walker, S. (2015). Fitting linear mixed-effects models using lme4. *Journal of Statistical Software*, *67*(1), 1–48. 10.18637/jss.v067.i01

[CR3] Castro, N., Mendoza, J. M., Tampke, E. C., & Vitevitch, M. S. (2018). An account of the Speech-to-Song illusion using node structure theory. *PLOS ONE*, *13*(6), e0198656. 10.1371/journal.pone.0198656 29883451 10.1371/journal.pone.0198656 PMC5993277

[CR4] Cutler, A., & Otake, T. (1999). Pitch accent in spoken-word recognition in Japanese. *The Journal of the Acoustical Society of America*, *105*(3), 1877–1888. 10.1121/1.426724 10089610 10.1121/1.426724

[CR5] Deutsch, D., Henthorn, T., & Lapidis, R. (2011). Illusory transformation from speech to song. *The Journal of the Acoustical Society of America*, *129*(4), 2245–2252. 10.1121/1.3562174 21476679 10.1121/1.3562174

[CR6] Dolan, T. (2006). Hiberno-English in transition. *Études Irlandaises*, *31*(2), 33–45.

[CR7] Douven, I. (2018). A bayesian perspective on likert scales and central tendency. *Psychonomic Bulletin & Review*, *25*(3), 1203–1211. 10.3758/s13423-017-1344-2 28752379 10.3758/s13423-017-1344-2

[CR8] Falk, S., & Rathcke, T. (2010). On the speech-to-song illusion: Evidence from German. In *Proceedings of the 5th International Conference on Speech Prosody* (Paper 169). ISCA. 10.21437/SpeechProsody.2010-262

[CR9] Falk, S., Rathcke, T., & Dalla Bella, S. (2014). When speech sounds like music. *Journal of Experimental Psychology: Human Perception and Performance*, *40*(4), 1491–1506. 10.1037/a0036858 24911013 10.1037/a0036858

[CR10] Groenveld, G., Burgoyne, J. A., & Sadakata, M. (2020). I still hear a melody: Investigating Temporal dynamics of the Speech-to-Song illusion. *Psychological Research Psychologische Forschung*, *84*(5), 1451–1459. 10.1007/s00426-018-1135-z 30627768 10.1007/s00426-018-1135-z

[CR11] Harnad, S. (1987). Psychophysical and cognitive aspects of categorical perception: A critical overview. In S. Harnad (Ed.), *In categorical perception: The groundwork of cognition* (pp. 1–25). Cambridge University Press.

[CR12] Hartig, F. (2024). *DHARMa: Residual diagnostics for hierarchical (multi-level / mixed) regression models* (Version 0.4.7) [R package]. https://CRAN.R-project.org/package=DHARMa

[CR13] Hickey, R. (2008). Irish English: Phonology. In B. Kortmann, C. Upton, & E. W. Schneider (Eds.), *The British Isles: A Handbook of Varieties of English* (pp. 71–104). De Gruyter Mouton. 10.1515/9783110208399.1.71

[CR14] Hiemstra, A., & Sadakata, M. (2024). Sentences used in the speech-to-song illusion: Comparisons of acoustic vowel space. *Musicae Scientiae*, *28*(3), 520–538. 10.1177/10298649231224786

[CR15] Jaisin, K., Suphanchaimat, R., Figueroa Candia, M. A., & Warren, J. D. (2016). The Speech-to-Song illusion is reduced in speakers of tonal (vs. Non-Tonal) languages. *Frontiers in Psychology*, *7*, 662. https://doi.org/10.3389/fpsyg.2016.00662. https://www.frontiersin.org/articles/27242580 10.3389/fpsyg.2016.00662 PMC4860502

[CR16] Kachlicka, M., Patel, A. D., Liu, F., & Tierney, A. (2024). Weighting of cues to categorization of song versus speech in tone-language and non-tone-language speakers. *Cognition*, *246*, 105757. 10.1016/j.cognition.2024.105757 38442588 10.1016/j.cognition.2024.105757

[CR17] Kallen, J. L. (2012). The english Language in ireland:an introduction. *International Journal of Language Translation and Intercultural Communication*, *1*, 25–41. 10.12681/ijltic.8

[CR18] Kawahara, S. (2015). The phonology of Japanese accent. In H. Kubozono (Ed.), *The handbook of Japanese phonetics and phonology* (pp. 445–492). De Gruyter Mouton.

[CR20] Kubit, B. M., Deng, C., Tierney, A., & Margulis, E. H. (2024). Rapid learning and long-term memory in the speech-to-song illusion. *Music Perception*, *41*(5), 348–359. 10.1525/mp.2024.41.5.348

[CR21] Kubozono, H. (2018). Pitch accent. In Y. Hasegawa (Ed.), *The Cambridge handbook of Japanese linguistics* (pp. 154–180). Cambridge University Press. 10.1017/9781316779194.010

[CR22] Kuhl, P. K. (1991). Human adults and human infants show a perceptual magnet effect for the prototypes of speech categories, monkeys do not. *Perception & Psychophysics*, *50*(2), 93–107. 10.3758/BF03212211 1945741 10.3758/bf03212211

[CR23] Kutas, M., & Hillyard, S. A. (1980). Reading senseless sentences: Brain potentials reflect semantic incongruity. *Science*, *207*(4427), 203–205. 10.1126/science.7350657 7350657 10.1126/science.7350657

[CR24] Kuznetsova, A., Brockhoff, P. B., & Christensen, R. H. B. (2017). LmerTest package: Tests in linear mixed effects models. *Journal of Statistical Software*, *82*(13), 1–26. 10.18637/jss.v082.i13

[CR25] Lenth, R. (2024). emmeans: Estimated marginal means, aka least-squares means (Version 1.10.2) [R package]. https://CRAN.R-project.org/package=emmeans

[CR26] Margulis, E. H. (2013). *On repeat: How music plays the Mind*. Oxford University Press. 10.1093/acprof:oso/9780199990825.001.0001

[CR27] Margulis, E. H., Simchy-Gross, R., & Black, J. L. (2015). Pronunciation difficulty, Temporal regularity, and the speech-to-song illusion. *Frontiers in Psychology*. 10.3389/fpsyg.2015.00048. 6. 25688225 10.3389/fpsyg.2015.00048 PMC4310215

[CR28] Mattock, K., & Burnham, D. (2006). Chinese and english infants’ tone perception: Evidence for perceptual reorganization. *Infancy*, *10*(3), 241–265. 10.1207/s15327078in1003_3

[CR29] McDermott, J. H., Schemitsch, M., & Simoncelli, E. P. (2013). Summary statistics in auditory perception. *Nature Neuroscience*, *16*(4), 493–498. 10.1038/nn.3347 23434915 10.1038/nn.3347 PMC4143328

[CR30] Mugitani, R., Kobayashi, T., Hayashi, A., & Fais, L. (2019). The use of pitch accent in Word–Object association by monolingual Japanese infants. *Infancy*, *24*(3), 318–337. 10.1111/infa.12279 32677192 10.1111/infa.12279

[CR31] Müllensiefen, D., Gingras, B., Musil, J., & Stewart, L. (2014). Measuring the facets of musicality: The goldsmiths musical sophistication index (Gold-MSI). *Personality and Individual Differences*, *60*(Supplement), S35. 10.1016/j.paid.2013.07.081

[CR32] R Core Team. (2023). *R: A Language and environment for statistical computing*. R Foundation for Statistical Computing. https://www.R-project.org/

[CR33] Rathcke, T., Falk, S., & Dalla Bella, S. (2021). Music to your ears: Sentence sonority and listener background modulate the speech-to-song illusion. *Music Perception*, *38*(5), 499–508. 10.1525/mp.2021.38.5.499

[CR34] Rowland, J., Kasdan, A., & Poeppel, D. (2019). There is music in repetition: Looped segments of speech and nonspeech induce the perception of music in a time-dependent manner. *Psychonomic Bulletin & Review*, *26*(2), 583–590. 10.3758/s13423-018-1527-5 30238294 10.3758/s13423-018-1527-5

[CR36] Sadakata, M. (2024). Exploring the speech-to-song transformation: linguistic influences in tonal and non-tonal language speakers. In L. Samuelson, S. Frank, M. Toneva, A. Mackey, & E. Hazeltine (Eds.), Proceedings of the 46th Annual Conference of the Cognitive Science Society. Cognitive Science Society. https://escholarship.org/uc/cognitivesciencesociety

[CR37] Sadakata, M. (2025, December 19). Pitch accent speakers exhibit Speech-to-Song transformation accompanied by reduced musicality ratings: A cross-linguistic study [OSF project]. OSF. https://osf.io/c37td 10.1007/s00426-026-02262-0 PMC12950085 41762264

[CR35] Sadakata, M., Yamaguchi, Y., Ohsawa, C., Matsubara, M., Terasawa, H., von Schnehen, A., Müllensiefen, D., & Sekiyama, K. (2023). The Japanese translation of the Gold-MSI: Adaptation and validation of the self-report questionnaire of musical sophistication. *Musicae Scientiae*, *27*(3), 798–810. 10.1177/10298649221110089

[CR38] Shport, I. A. (2015). Perception of acoustic cues to Tokyo Japanese pitch-accent contrasts in native Japanese and Naive english listeners. *The Journal of the Acoustical Society of America*, *138*(1), 307–318. 10.1121/1.4922468 26233031 10.1121/1.4922468

[CR39] Shport, I. A. (2016). Training english listeners to identify pitch-accent patterns in Tokyo Japanese. *Studies in Second Language Acquisition*, *38*(4), 739–769. 10.1017/S027226311500039X

[CR40] Simchy-Gross, R., & Margulis, E. H. (2018). The sound-to-music illusion: Repetition can musicalize nonspeech sounds. *Music & Science*, *1*, 2059204317731992. 10.1177/2059204317731992

[CR41] Smit, E. A., & Rathcke, T. V. (2024). Beyond musical training: Individual influences on the perception of the speech-to-song illusion. *Psychology of Music*, *53*(4), 507–522. 10.1177/03057356241256652

[CR42] Soehlke, L. E., Kamat, A., Castro, N., & Vitevitch, M. S. (2022). The influence of memory on the speech-to-song illusion. *Memory & Cognition*, *50*(8), 1804–1815. 10.3758/s13421-021-01269-9 35083717 10.3758/s13421-021-01269-9 PMC9767999

[CR43] Sugiyama, Y. (2008). Production and perception of pitch accent in Japanese. *Annual Meeting of the Berkeley Linguistics Society*, 317–328. 10.3765/bls.v34i1.3579

[CR44] Tamaoka, K., Saito, N., Kiyama, S., Timmer, K., & Verdonschot, R. G. (2014). Is pitch accent necessary for comprehension by native Japanese speakers? – An ERP investigation. *Journal of Neurolinguistics*, *27*(1), 31–40. 10.1016/j.jneuroling.2013.08.001

[CR45] Tiedemann, F. (2022). *gghalves: Compose half-half plots using your favourite geoms* (Version 0.1.4) [R package]. https://CRAN.R-project.org/package=gghalves

[CR46] Tierney, A., Dick, F., Deutsch, D., & Sereno, M. (2013). Speech versus song: Multiple pitch-sensitive areas revealed by a naturally occurring musical illusion. *Cerebral Cortex*, *23*(2), 249–254. 10.1093/cercor/bhs003 22314043 10.1093/cercor/bhs003 PMC3539450

[CR47] Tierney, A., Patel, A. D., & Breen, M. (2018a). Acoustic foundations of the speech-to-song illusion. *Journal of Experimental Psychology: General*, *147*(6), 888–904. 10.1037/xge0000455 29888940 10.1037/xge0000455

[CR48] Tierney, A., Patel, A. D., & Breen, M. (2018b). Repetition enhances the musicality of speech and tone stimuli to similar degrees. *Music Perception*, *35*(5), 573–578. 10.1525/mp.2018.35.5.573

[CR49] Tierney, A., Patel, A. D., Jasmin, K., & Breen, M. (2021). Individual differences in perception of the speech-to-song illusion are linked to musical aptitude but not musical training. *Journal of Experimental Psychology: Human Perception and Performance*, *47*(12), 1681–1697. 10.1037/xhp0000968 34881953 10.1037/xhp0000968

[CR50] Vanden, B., Nederlanden, C. M., Hannon, E. E., & Snyder, J. S. (2015). Everyday musical experience is sufficient to perceive the speech-to-song illusion. *Journal of Experimental Psychology: General*, *14*(3), e43–e49. 10.1037/xge0000056 10.1037/xge0000056 25688906

[CR53] VandenBosch der Nederlanden, C. M., Hannon, E. E., & Snyder, J. S. (2015). Everyday musical experience is sufficient to perceive the speech-to-song illusion. *Journal of experimental psychology. General, 144*(2), e43–e49. 10.1037/xge0000056 10.1037/xge0000056 25688906

[CR52] Vet, D. J. (n.d.). ED - Experimental Designer [Computer software]. https://www.fon.hum.uva.nl/dirk/

[CR51] Wickham, H. (2016). *ggplot2: Elegant graphics for data analysis*. Springer-. https://ggplot2.tidyverse.org

