# Supplementary material for: Pitch accent speakers exhibit speech-to-song transformation accompanied by reduced musicality ratings: a cross-linguistic study
Source: Psychol Res. 2026 Feb 28;90(2):45. doi: 10.1007/s00426-026-02262-0 (PMC12950085; doi:10.1007/s00426-026-02262-0)
Supplement: Supplementary file 1 — Supplementary Material 1 (DOCX 332 KB) [file 426_2026_2262_MOESM1_ESM.docx]

**Supplementary Information (Table S1, Table S2, Table S3, Figure S1)**

**Pitch accent speakers exhibit Speech-to-Song transformation accompanied by reduced musicality ratings: A cross-linguistic study**

**Psychological Research**

**Authors**

Makiko Sadakata^1^, Martha Nobbe Smyth^2^, Marijn van ’t Veer^3^, Akihiro Tanaka^4^

**Affiliations:**

^1^Institute for Logic, Language and Computation / Musicology Department, University of Amsterdam

^2^Research Master Brain and Cognitive Sciences, University of Amsterdam

^3^Lingustics Department, University of Amsterdam / Centre for Linguistics, Leiden University

^4^Cognitive Psychology Department, Tokyo Woman’s Christian University

**Corresponding author:**

**Makiko Sadakata**

Institute for Logic, Language and Communication, University of Amsterdam, The Netherlands

Science Park 107, 1098XG Amsterdam

**Corresponding author's Email address:**

m.sadakata@uva.nl

**Table S1: Model Comparison for the absolute songlikeness analysis.** Comparison between the simplified and full linear mixed-effects models. The table reports the number of estimated parameters (npar), Akaike Information Criterion (AIC), Bayesian Information Criterion (BIC), log-likelihood (logLik), deviance, the likelihood ratio test statistic (χ²), and degrees of freedom (df). The comparison indicates no significant improvement in model fit for the full model (*p* = 0.51).

| **Model** | **npar** | **AIC** | **BIC** | **logLik** | **Deviance** | **χ2** | **df** |
| --- | --- | --- | --- | --- | --- | --- | --- |
| Simplified Model | 12 | 12268 | 12341 | -6122 | 12244 | - | - |
| Full Model | 14 | 12271 | 12356 | -6121.3 | 12243 | 1.3408 | 2 |

**Simulation-based residual diagnostics and sensitivity analyses (Figure S1, Table S2)**

Model assumptions were evaluated using simulation-based residual diagnostics implemented in the DHARMa package. Residual diagnostics were based on 1000 simulations. Visual inspection of the DHARMa diagnostic plots showed no evidence of systematic deviations in residual dispersion, consistent with the non-significant dispersion test (p = .88). Tests of residual uniformity and outliers were significant; however, deviations from uniformity were modest and primarily confined to the distribution tails. Such patterns are commonly observed when Gaussian linear mixed-effects models are applied to Likert-scale data with large sample sizes.

**Figure S1. Simulation-based residual diagnostics generated using the DHARMa package.** The left panel shows the quantile–quantile plot of simulated residuals against the expected uniform distribution. The right panel shows DHARMa residuals plotted against model predictions

To assess the robustness of the results to extreme observations, we conducted a sensitivity analysis excluding the most extreme 1% of scaled DHARMa residuals. Extreme observations were rare and not concentrated in any specific combination of participant group, stimulus language, or response type. The linear mixed-effects model was refit using the same specification as in the main analysis. All key fixed effects, including the STS transformation effect and the Group × Stimulus Language interactions, showed the same direction and qualitative interpretation as in the full dataset. Estimated effect sizes differed only modestly between the original and trimmed models, indicating that the main results were not driven by a small number of extreme observations. Table S2 summarizes the comparison of fixed-effect estimates between the original and sensitivity models.

**Table S2. Summary of the comaprison between the original and sensitivity models.**

| **term** | **estimate_original** | **estimate_trimmed** | **estimate_diff** |
| --- | --- | --- | --- |
| (Intercept) | 2.929981061 | 2.787617577 | -0.142363484 |
| GroupDU | -0.419375 | -0.349821343 | 0.069553657 |
| GroupDU:LanguageJapanese | 1.647916667 | 1.701487488 | 0.053570822 |
| GroupEN | -0.219024123 | -0.150016212 | 0.069007911 |
| GroupEN:LanguageJapanese | 2.367214912 | 2.45506996 | 0.087855047 |
| LanguageJapanese | -1.322462121 | -1.314066784 | 0.008395338 |
| ResponseTypeFinal | 1.969204545 | 2.235719377 | 0.266514831 |
| ResponseTypeFinal:GroupDU | 0.139583333 | -0.004304676 | -0.143888009 |
| ResponseTypeFinal:GroupEN | -0.756030702 | -0.926295148 | -0.170264446 |
| ResponseTypeFinal:LanguageJapanese | -0.584242424 | -0.726282435 | -0.14204001 |

**Table S3. Correlations between Gold-MSI (musical sophistication) scores and songlikeness outcome measures by group and stimulus language.** This table reports Pearson correlation coefficients (r) between participants’ Gold-MSI scores and three outcome measures—Initial rating, Final rating, and Transform score (Final minus Initial)—calculated separately for each language group (JP, DU, EN) and for English and Japanese stimuli. P-values are presented both uncorrected and following false discovery rate (FDR).

| **Group** | **Stimulus Language** | **Variable** | **Correlation coefficient** | **p-value (FDR adjusted)** |
| --- | --- | --- | --- | --- |
|  |  | Initial | 0.44 | 0.137 |
|  | English | Final | 0.59 | 0.051 |
| JP |  | Transform | 0.30 | 0.257 |
|  |  | Initial | 0.18 | 0.510 |
|  | Japanese | Final | 0.48 | 0.176 |
|  |  | Transform | 0.41 | 0.176 |
|  |  | Initial | 0.56* | 0.020 |
|  | English | Final | 0.54* | 0.020 |
| DU |  | Transform | -0.03 | 0.888 |
|  |  | Initial | 0.63** | 0.008 |
|  | Japanese | Final | 0.42* | 0.010 |
|  |  | Transform | -0.23 | 0.322 |
|  |  | Initial | 0.02 | 0.939 |
|  | English | Final | -0.35 | 0.212 |
| EN |  | Transform | -0.48 | 0.115 |
|  |  | Initial | 0.34 | 0.228 |
|  | Japanese | Final | -0.02 | 0.926 |
|  |  | Transform | -0.68** | 0.004 |
